# The Development of Oral Amphotericin B to Treat Systemic Fungal and Parasitic Infections: Has the Myth Been Finally Realized?

**DOI:** 10.3390/pharmaceutics11030099

**Published:** 2019-02-26

**Authors:** Grace Cuddihy, Ellen K. Wasan, Yunyun Di, Kishor M. Wasan

**Affiliations:** 1 College of Pharmacy and Nutrition, University of Saskatchewan, Saskatoon, SK S7N 2Z4, Canada; cac579@mail.usask.ca (G.C.); ellen.wasan@usask.ca (E.K.W.); yud083@mail.usask.ca (Y.D.); 2 Faculty of Pharmaceutical Sciences, University of British Columbia, Vancouver, BC V6T 1Z3, Canada

**Keywords:** oral formulation, amphotericin B, fungal infections, parasitic infections, developing world, drug delivery

## Abstract

Parenteral amphotericin B has been considered as first-line therapy in the treatment of systemic fungal and parasitic infections, however its use has been associated with a number of limitations including affordability, accessibility, and an array of systemic toxicities. Until very recently, it has been very challenging to develop a bioavailable formulation of amphotericin B due to its physical chemical properties, limited water and lipid solubility, and poor absorption. This perspective reviews several novel oral Amphotericin B formulations under development that are attempting to overcome these limitations.

## 1. Preamble

One of the authors (KMW) was presenting grand rounds to the infectious disease group at Vancouver General Hospital, discussing combination therapies to treat systemic fungal infections, particularly those patients who were about to go through organ transplantation. An infectious disease physician asked if it was possible to develop an oral formulation of amphotericin B to treat patients. This physician commented that if an oral formulation could be developed, then it would be widely used, because it would have the potential to overcome many of the limitations of intravenous administration. These limitations include affordability, accessibility, and the well-known systemic toxicities associated with amphotericin B. At the time, KMW considered it extremely challenging to develop a bioavailable formulation of amphotericin B that would achieve the tissue concentrations required to have a pharmacological effect and ameliorating the dose-dependent nephrotoxicity associated with the drug. Factors include the large molecular weight of amphotericin B, its amphoteric physical chemical nature, very poor water and lipid solubility, as well as acid lability. 

However, as KMW thought about it, it became clear that with a growing understanding of dietary and excipient lipid processing in the gastrointestinal tract (GIT) as well as associated new drug delivery technologies, it could in fact be possible to develop an efficacious oral formulation.

## 2. Purpose

The aim of this perspective is firstly to provide sufficient background information on both amphotericin B (AmB) and the target disease leishmaniasis, as well as to explain the need for an oral formulation of this life-saving medication. Secondly, our purpose is to describe pharmaceutical advances that have led to several novel AmB formulations which have emerged over the last decade. Equally important is to discuss the role of formulation in reducing specific barriers to treatment in highly endemic regions of visceral leishmaniasis, such as cost and storage considerations.

## 3. Chemistry of AmB

### 3.1. Structure Overview

**Figure 1:**
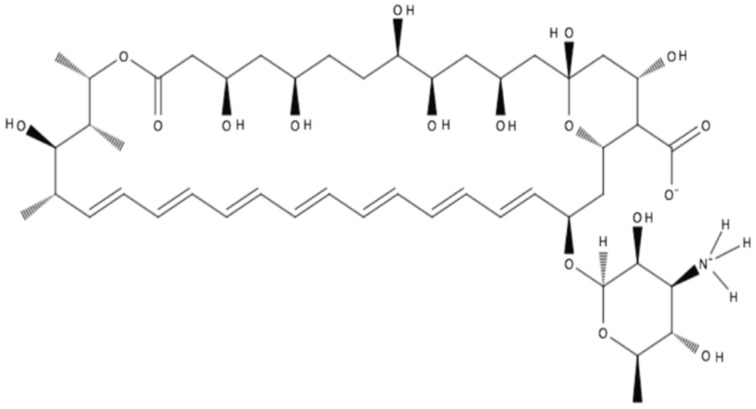
Structure of amphotericin B in zwitterionic form.

AmB has a large, highly complex structure (Figure 1). It is classified as a polyene macrolide antibiotic; specifically, it is known as a macrolide because it contains a polyketide that is linked to a mycosamine sugar. Furthermore, it is classified as a polyene macrolide due to the presence of the hydrophobic polyene subunit, which is attached to the hydrophilic polyol portion of the molecule [1]. Overall, it consists of a 38-membered macrolactone ring, which is β-glycosylated with mycosamine at the C-19 hydroxyl position [1]. Seven conjugated double bonds comprise the polyene subunit, while an ester and a ketone separated by 12 carbons and substituted with six hydroxyl groups comprise the polyol subunit of the molecule. 

### 3.2. Structure-Activity Relationship

The structure-activity relationship of AmB has been the focus of numerous studies over the last three decades, which are briefly outlined below. Generalizations can be made with regard to the pharmacophore of this molecule: The positively charged amino group is required for activity; the polyene subunit is important for activity; if the carboxyl group is negatively charged, it leads to decreased selectivity for ergosterol over cholesterol; and conversely, N-aminoacylation leads to improved selectivity [1]. 

### 3.3. Mechanism of Action of AmB

Polyene antifungals such as AmB act by binding with ergosterol of the fungal cell walls and forming pores which permit leakage of cell contents, which eventually results in apoptosis [2,3]. This binding of AmB to ergosterol occurs through hydrophobic interactions, disrupting the lipid membrane integrity and resulting in the formation of pores [4,5]. These channels in the cell membrane allow efflux of small ions and other macromolecules, such as potassium and magnesium [6,7]. Simulation of the AmB–ergosterol structure finds that the formed pores promote water transport across the cell membrane, which might further disrupt the intracellular environment [8]. Recent evidence indicates that ergosterol binding and pore formation may not be the only mechanism leading to fungal cell death. It has been reported that AmB could kill yeasts by extracting ergosterols from cell membrane lipid layers [9,10]. Furthermore, it has been proposed that AmB causes accumulation of intracellular reactive oxygen species (ROS), which also contributes to the antifungal effect of this drug [11]. Several studies have described elevated ROS in fungal cells treated with AmB [12,13]. However, it is still not clear how AmB induces ROS production. 

### 3.4. Bioavailability of AmB

The complexity of the AmB molecule is partly due to the individual functional groups present but also due to the asymmetry of the important subunits that make up the molecule. For instance, the polyol subunit is highly hydrophilic with many hydrogen bond donors and acceptors available to interact with molecules of water. By contrast, the polyene subunit is highly hydrophobic, as it consists of seven conjugated double bonds in a hydrocarbon chain 14 carbons in length. In addition to the amphiphilic nature of AmB, it also has a zwitterionic character on one portion of the molecule with the carboxylic acid and primary amine functional groups, which can be negatively and positively charged, respectively [14]. Therefore, overall, this asymmetric, amphiphilic molecule with zwitterionic character demonstrates a poor aqueous solubility of less than 1 mg/L at physiological pH, leading to its precipitation in aqueous media [14]. Lipinski’s rule of five, which describes drug features that increase the probability of oral bioavailability based on passive diffusion though cellular membranes, can be applied to AmB with predictable results. AmB violates three out of four rules: AmB has more than 5 H-bond donors, more than 10 H-bond acceptors, and a molecular weight greater than 500 Da [14]. Thus, AmB will not easily be absorbed through the gastrointestinal mucosal membranes by passive transport following oral administration, which is confirmed by AmB’s known low oral bioavailability of 0.2–0.9% [14]. Together with the aforementioned chemical complexities of AmB, there are significant barriers that must be overcome for an oral formulation of AmB to be developed. 

## 4. Treating Visceral Leishmaniasis (VL)

Leishmaniasis is one of 20 conditions listed in the World Health Organization (WHO)’s list of “Neglected Tropical Diseases” [15]. Despite having effective treatments for the various presentations of the infection since the late 1950s, leishmaniasis is still a major concern in the 74 endemic countries identified by the WHO’s Global Health Observatory data repository in 2016 [16]. Although the distribution of the disease is quite widespread, the large majority of new cases are limited to the following hyperendemic regions: Brazil, Ethiopia, India, Kenya, Somalia, South Sudan, and Sudan [17,18]. In 2016 alone, the number of reported cases of VL (or kala-azar), the most severe form of the disease, was: 6249 in India; 4285 in South Sudan; 3810 in Sudan; 3200 in Brazil; 1593 in Ethiopia; and 911 in Somalia [19]. 

Leishmaniasis is a vector-borne parasitic protozoan infection caused by more than 20 species of the *Leishmania* genus [20]. The known vectors of these parasites are the female sand flies of the genus *Phlebotomus*, which have a broad geographical distribution ranging from areas of the tropics, subtropics, and even temperate regions [21]. Additionally, domestic dogs are known reservoir hosts in the Mediterranean and New World regions [17].

*Leishmania* has a digenetic life cycle, switching between sand fly stages and human stages transmitted by sand flies biting humans. During a blood meal, the metacyclic promastigotes inoculated into the human skin immediately invade into macrophages, dendritic cells, fibroblasts, and keratinocytes and subsequently deactivate the host’s complement system, suppressing the production of microbicidal molecules, such as superoxide and nitric oxide [22,23,24]. Although the parasites are found in these various cell types, macrophages are the main host cells where the metacyclic promastigotes differentiate into amastigotes [25]. The amastigotes continue to proliferate and disseminate into other tissues and organs, including the liver, spleen, and bone marrow. The existence of cutaneous or visceral leishmaniasis symptoms in humans depends on the parasite species, host conditions, and other factors [20]. At this point, if the sand fly bites the infected host again, the circulating amastigote-infected macrophages are likely to transmit to the new vector. In the gut of sand flies, amastigotes transformed into extracellular promastigotes, which takes approximately 7–14 days for transmissible infection to develop in the vector [26]. The promastigotes then migrate anteriorly to the stomodeal valve of the sand fly and undergo a series of developmental transitions to form infectious metacyclic promastigotes. Finally, during a blood meal on an appropriate host, a new digenetic cycle of leishmaniasis will begin.

### 4.1. Amphotericin B Parenteral Formulations

Amphotericin B (AmB) is a polyene macrolide antibiotic administered parenterally in the treatment of a variety of systemic fungal infections including candidiasis, aspergillosis, fusariosis, and zygomycosis [27]. In addition, AmB has exhibited antiparasitic activity for certain protozoan infections, including leishmaniasis as well as primary amoebic meningoencephalitis [28]. Prior to the development of lipid based formulations, the commercially available formulation used in the clinic was Fungizone®, a conventional micellar form of AmB in a complex with deoxycholate [29]. Unfortunately, the conventional form is associated with renal toxicity, which led to the development of other nonconventional formulations [30]. Nonconventional or lipid-based formulations have been developed to overcome some of the toxicity problems associated with the conventional formulation. There are several lipid-based parenteral formulations which have been marketed to treat fungal infections, which include the liposomal formulation AmBisome^®^, the lipid complex formulation Abelcet^®^, and a colloidal dispersion formulation Amphocil^®^ (Amphotec) [31,32,33]. More recently, an emulsion form of AmB (Amphomul^®^) was developed and completed its Phase III clinical trial in 2014 [34]. The aim of this trial was to assess the safety and efficacy of the parenteral lipid emulsion formulation compared to AmBisome^®^ as a single infusion treatment for VL [34]. Overall, the drawbacks of the conventional parenteral formulation are the administration route, treatment duration, infusion time, and most importantly, the toxicities associated with treatment. It is, however, still widely used in developing nations where patients do not have access to the safer yet more expensive nonconventional formulations [27]. 

### 4.2. Visceral Leishmaniasis Treatment Options and Limitations

Over the past few decades, treatment for VL is limited to pentavalent antimonials, AmB deoxycholate and pentamidine, and more recently, liposomal AmB, mitefosine, and paromomycin [35]. At present, in developed countries, the first-line therapy for VL in both immunocompetent and immunocompromised patients is short-course intravenous liposomal AmB, which has been demonstrated to have improved efficacy with reduced nephrotoxicity compared to conventional formulations [36]. However, more than 90% of global VL cases occur in developing countries, where conventional AmB is still considered first-line therapy for VL because it is the most affordable option [37,38]. An oral formulation of AmB would improve access to safe and effective treatment for VL in these affected regions worldwide by removing the barriers of high costs, the need for hospitalization, and a requirement for cold chain transport and storage conditions. 

Cost of treatment is an important consideration for most patients; since liposomal AmB is 30 times more expensive than the conventional formulation, it is a huge limitation for patients in developing countries [17]. In 2010, the WHO released the “Costs of medicines in current use for the treatment of leishmaniasis” that included drug prices per unit and their estimated prices per VL treatment [39]. This document has the price per unit provided by the manufacturer, or the WHO-negotiated prices where applicable. They stated that the median cost per 50 mg of AmB deoxycholate to be $7.5 USD in comparison to the WHO negotiated price of $18 USD per 50 mg vial of AmBisome^®^. The estimated price per VL treatment was $252 USD per 2–4 day treatment with 20 mg/kg AmBisome^®^ in comparison to $20 USD for a 30 day treatment (alternating days) with 1 mg/kg AmB deoxycholate [39]. However, these estimates were done for a patient weighing 35 kg (or 77 lbs); therefore, many patients’ treatment would be appreciably more expensive. Moreover, the treatment regimen used for the estimation includes a shorter treatment duration than what is recommended, as previously described. It remains unclear which guidelines WHO used to determine the treatment regimen as it was not disclosed, and standard treatment regimens will vary by country. If the manufacturer recommendations for treatment duration were used, the estimated cost of treatment would undoubtedly increase. A reduction in the cost of treatment, in the form of an oral formulation of AmB, would greatly improve access to treatment for those where the financial burden of treatment is simply unreasonable.

Additionally, parenteral formulations must be administered in a hospital setting under the supervision of health care professionals. Beyond the direct costs of in-patient treatment, including admission, medical supplies, and charges for physician and laboratory services, there are numerous indirect costs which make this form of treatment impossible for many low-income populations [17,40]. Indirect costs may include: Travel to the healthcare facility, food for the patient and caregiver while in hospital, loss of income of the patient and/or their family members which accompany them, as well as any other unforeseen miscellaneous costs [17]. Oral AmB would permit out-patient treatment where patients could stay in their rural communities for the duration of their treatment, reducing the economic burden of in-patient health care costs and the detrimental indirect costs of treatment for patients and their families.

Although nonconventional formulations have improved the safety profile of AmB, there are some inherent drawbacks to using a parenteral formulation of any kind in the developing nations which are most affected by VL. The storage and transportation of liposomal AmB is a limitation, as the intact vials must be stored ≤25 °C and reconstituted vials are only stable for 24 h in 2–8 °C [41]. This is an important limitation if one considers that all of the hyperendemic regions occur in tropical or subtropical climates where proper refrigeration may not be feasible. In general, compared with parenteral formulations, oral dosage forms are more flexible with their required storage conditions in terms of temperatures and sterility, making them an attractive alternative. Another important limitation is the different aggregation states of AmB. This amphipathic molecule has the ability to self-aggregate in aqueous solution, which affects the safety profile of the different formulations of this drug [42]. For instance, the monomeric form of AmB remains the safest due to its ability to target ergosterol; thus, many formulations attempt to deliver AmB to target tissues in this form [42,43,44]. Conversely, the dimeric form of AmB, which is the most common state of reconstituted Fungizone^®^, is associated with the worst toxicity of AmB [42,45]. Furthermore, the poly-aggregated state is safer than the dimeric form [46,47].

## 5. Oral Formulations of AmB Currently in Development

### 5.1. Solid Lipid Nanoparticles

Chaudhari et al. (2015) developed solid lipid nanoparticles (SLNs) loaded with AmB (AmbiOnp) to overcome the poor oral bioavailability and kidney toxicity issues with AmB [47] (Table 1). The authors argued that producing a formulation that keeps AmB in its monomeric and/or super-aggregated form will keep the drug in a form which preferentially targets ergosterol, as opposed to its dimeric or oligomeric form, with its high affinity for cholesterol, which is responsible for the toxicity associated with conventional AmB. The AmbiOnp formulation was prepared by a probe sonication-assisted nanoprecipitation technique which produced a greater proportion of super-aggregated AmB that accumulated to a lesser extent in the kidneys, as reported in in biodistribution studies. This oral formulation was found to have a greatly improved safety profile compared to conventional IV-administered Fungizone^®^, with kidney tissue concentrations of approximately 84.5 ± 22.9 ng/g and 518.6 ± 31.5 ng/g, respectively, eight hours following administration [47]. Furthermore, the authors did not report any adverse reactions with the new formulation. In vivo pharmacokinetic studies demonstrated that orally administered AmbiOnp had a 1.05 relative bioavailability compared to intravenous Fungizone^®^, the long-standing gold standard of therapy for systemic fungal infections and VL. AmbiOnp demonstrated an optimal sustained release of AmB from the SLN delivery system, with 60% encapsulated in simulated intestinal fluid (SIF) over a period of 6 h. This formulation had the added benefit of an improved stability profile for storage conditions compared to conventional AmB:AmbiOnp was shown to be stable in 2–8 °C for 3 months or around 15 days when stored at 25 °C and 40 °C [47]. 

### 5.2. PLGA–PEG Nanoparticles

In contrast to the super-aggregate form of AmbiOnp, Radwan et al. (2017) formulated a nanoparticle formulation in the hopes that it would release AmB solely in its monomeric form [43] (Table 1). This formulation consisted of a poly(lactide-*co*-glycolide)–poly(ethylene glycol) (PLGA–PEG) copolymer loaded with AmB and glycyrrhizic acid as an absorption enhancer. This delivery system was formulated in hopes of an increase in the solubility of AmB, lessened toxicity, and the delivery of monomeric AmB to ensure the efficacy of the formulation. In vivo efficacy was not investigated; however, in vitro investigations found that the PLGA–PEG formulation had a greater antifungal activity with a minimum inhibitory concentration (MIC) reduction of fourfold or greater than that of Fungizone^®^ 24 and 48 h after inoculation with *Candida albicans* in rats [43]. Pharmacokinetic studies found that the formulation had a 1.3 relative bioavailability compared to Fungizone^®^ [43]. Kumar et al. (2015) also developed a PLGA–PEG encapsulated AmB formulation and tested the efficacy against *Leishmania donovani* in hamsters [48]. According to the report, this formulation was able to inhibit the parasite load in the liver by 93.2% compared to the free Amb group (74.6%) [48].

### 5.3. Chitosan-Coated Nanostructured Lipid Carriers

Ling Tan et al. (2018) designed a formulation consisting of a mixture of solid lipids and lipid oils, which they called nanostructured lipid carriers (NLC), with added chitosan coating for mucoadhesion [42] (Table 1). The authors’ aim was to maximize lymphatic transport of their formulation to improve the oral bioavailability of AmB. Additionally, this formulation aimed to deliver AmB in its less toxic monomeric form. By one of the preparation methods tested, both the uncoated and chitosan coated NLC formulations were found to be stable in a predominantly monomeric form and, to a lesser extent, a poly-aggregate form for a 120-day period [42]. Encapsulation efficiency of the AmB-NLC formulation was 83.4 ± 0.72% and with a drug loading of 12.3 ± 0.11%, with the encapsulation efficiency significantly increasing with the chitosan coated form [42]. Both coated and uncoated forms demonstrated a biphasic release profile: An initial burst release phase followed by sustained release. The authors concluded that their formulation addressed the concerns of toxicity by keeping AmB in its monomeric and polyaggregated forms and that it has the potential to improve AmB’s oral bioavailability due to the mucoadhesive properties of the NLCs which permit uptake in the small intestine. They plan to follow up with in vivo pharmacokinetic studies and safety studies to confirm their findings [42].

### 5.4. Lecithin-Based Mixed Polymeric Micelles

Chen et al. (2015) prepared a self-assembling lecithin-based mixed polymeric micellar formulation as an oral delivery system of AmB [49] (Table 1). This micellar formulation uses lecithin as the lipid component with a number of polymers (including but not limited to Pluronic^®^ and 1,2-distearoyl-*sn*-glycero-3-phosphoethanolamine-*N*-methoxy(poly(ethylene glycol)-2000 (DSPE–PEG2K ), which are loaded with AmB using a thin film method. Specifically, the authors’ optimal formulation, which they named Ambicelles, consisted of AmB:lecithin:DSPE–PEG2K in a 1:1:10 mass ratio. Ambicelles were shown to increase the solubility of AmB from 0.001 to 5 mg/mL in addition to an improved relative oral bioavailability of 1.50 compared to that of Fungizone^®^ in rats, which the authors attributed to the optimal sustained delivery of monomeric AmB. In vitro cytotoxicity studies showed that Ambicelles were less cytotoxic than Fungizone^®^ and free AmB in a human colon adenocarcinoma cell line (HT29) [49].

### 5.5. O/W Microemulsion 

Another approach to the oral delivery of AmB is in the form of an oil-in-water microemulsion (O/W ME) [50] (Table 1). Silva et al. (2013) prepared an O/W ME using a surfactant mixture of Tween 80^®^ and Span 80^®^ with a hydrophilic–lipophilic balance of approximately 13 and an oil phase consisting of Capryol^®^ 90 or Capryol^®^ PGMC. This ME formulation was able to increase the solubility of AmB 1000-fold when compared to the aqueous solubility of AmB as well as providing favorable rheological behavior for an oral delivery system. Time-dependent cytotoxicity results found the ME formulation to be slightly less toxic than AmB in DMSO at concentrations up to 25 µg/mL in a murine macrophage cell line [50]. The authors attributed this time-dependent toxicity to the discovery of the formation of AmB aggregates which must be addressed before the development of this formulation progresses [50].

### 5.6. Pickering Emulsion

In contrast to the traditional emulsion, a Pickering emulsion uses solid particles instead of surfactants or other emulsifiers in order to stabilize its internal phase [51]. Richter et al. (2018) formulated an AmB-loaded Pickering emulsion stabilized by self-assembled cashew tree gum grafted with polylactide nanoparticles [51] (Table 1). The results demonstrated a novel formulation which permitted the incorporation of this poorly water-soluble drug into their emulsions with a process efficiency of up to approximately 47% and without suboptimal aggregation of the drug, as seen in some commercial preparations. The authors plan to continue the development of this formulation with subsequent in vitro release and toxicity studies [51].

### 5.7. Tragacanth/Acrylic Acid Copolymer

Mohamed et al. (2017) prepared a hydrogel drug carrier consisting of tragacanth and acrylic acid (Aac) using gamma-irradiation [52] (Table 1). This pH-sensitive copolymer formulation was shown to protect the AmB in an aggregated form in simulated gastric fluid (pH = 1) while drug was released as the formulation dissociated in SIF (pH = 7). The authors suggested that the release rate and total amount of drug released was dependent on pH and the Aac content of the copolymer with the aforementioned variables increasing with Aac content. In vivo antifungal efficacy investigations against candidiasis in mice showed that the oral (Trag/Aac)–AmB formulation (dose equivalent to 1 mg/kg) resulted in 0% mortality compared to the 10% mortality eight days post intravenous inoculation when administered intravenously with free AmB (1 mg/kg). Oral administration of (Trag/Aac)–AmB had similar efficacy to that of free AmB as shown by the measured reduction of colony forming units (CFU) found in kidney and liver tissues; free AmB reduced CFUs by 93% in the kidneys and 95% in the liver, comparatively (Trag/Aac)–AmB reduced CFUs by 97% and 93%, respectively [52]. Moreover, assessment of serum antibodies against *C. albicans* found no significant difference between the formulation of interest and free AmB, thus providing further evidence of the comparable efficacy of the (Trag/Aac)–AmB formulation. Furthermore, the authors did not find that that their formulation produced significant levels of the cytokines: Tumor necrosis factor-αβ, interleukin-1β, and nitric oxide in the kidney and the liver when compared to the free AmB-treated animals, which they interpreted as evidence supporting the superior safety of their formulation. In vivo toxicity investigations found (Trag/Aac)–AmB to be relatively safe, with negligible reported nephrotoxicity as demonstrated by no significant increase in creatinine or blood urea nitrogen (BUN) levels when compared with the AmB-treated control. Similar results were reported for liver toxicity as measured by serum aspartate aminotransferase and alanine aminotransferase enzymes. Further histopathological examinations were completed by the authors, which demonstrated that their oral formulation caused minimal renal damage and notable reduction of injury of hepatocytes when compared with the degenerative effects following treatment with free AmB on the renal glomerular tuft and hepatocyte necrosis [52].

### 5.8. Chitosan (CS) and Porphyrin (POR) Polymeric Nanocarrier

Bhatia et al. (2014) suggested that loading AmB as a polyelectrolyte complex into a biodegradable polymeric nanocarrier is an optimal solution to the delivery of this problematic drug [53] (Table 1). Specifically, they chose to use chitosan and porphyrin as two oppositely charged polymers with AmB associated with them. Stability studies showed that their polyelectrolyte complex formulation (i.e., with or without tripolyphosphate as a crosslinking agent) showed less degradation in simulated gastric fluid and a superior release profile for up to 12 h, when compared to plain AmB and chitosan-only nanoparticles. Moreover, an in vitro antifungal activity study found the formulated nanoformulations to yield significantly higher antifungal activity, as measured by their IC_50_, than the marketed formulations (AmB, Fungizone^®^, and AmBisome^®^) in the chosen fungal strains: *Aspergillus. fumigatus*, *Aspergillus. niger*, *Aspergillus. flavus*, and *Candida. albicans*. The most effective formulation was found to be the CS–POR–AmB formulation, with 23-fold greater activity than Ambisome^®^ in *A. fumigatus* and 12- to 15- fold greater activity in *A. niger*, *A. flavus*, and *C. albicans*. An in vitro hemolytic study found the authors’ nanoformulations to have less hemolytic toxicity than plain AmB and the chosen marketed formulations, proving the polyelectrolyte complexation (PEC) ormulation to be nontoxic up to concentrations of 55.5 μg/mL and with only approximately 4.1% hemolysis in the CS–POR–AmB formulation (compared to ~39.9% for plain AmB). However, the investigation into the in vivo toxicity of their POR formulations discovered an unexpected increase in platelet count and minimal decrease in red blood cell count, white blood cell count, hemoglobin, and hematocrit values when compared to the control. The authors suggested the platelet activation response may be due to the high sulfur content or due to the high anhydrogalactose (AGR) per mole concentration in their samples [53]. In vivo toxicity studies based on serum creatinine and blood urea nitrogen levels found that the renal toxicity at maximum dose was worst for Fungizone^®^ followed by CS–AmB, CS–POR–AmB, CS, and lastly POR. The authors proposed this result may be due to the associated release rate of each formulation as the AGR and sulfur present in POR produce a gelling effect which may be better suited for a gastroretentive release of AmB [53]. 

### 5.9. Chitosan–Ethylenediamine Tetraacetic Acid (EDTA) Microparticles 

Singh et al. (2013) characterized a novel solid self-nanoemulsifying drug delivery system (S-SNEDDS) formulation of AmB using spray dried covalently crosslinked EDTA–chitosan (COECH) microparticles for oral administration [54] (Table 1). They synthesized and characterized this formulation in hopes of developing an adequate delivery system for poorly water soluble and thermolabile drugs, such as AmB [54]. The authors reported that their formulation was indeed able to self-nanoemulsify into a thermodynamically stable delivery system once in contact with an aqueous environment. This formulation demonstrated a 12-fold improvement in in vitro dissolution relative to pure AmB. Overall, the authors concluded that their COECH–S-SNEDDS formulation prepared by spray drying technology was a reasonable approach which provided a solid substrate for the development of an AmB nanoemulsion for oral administration [54].

### 5.10. Carbon Nanotubes 

Prajapati et al. (2012) used carbon nanotubes (CNTs), which they covalently attached AmB in order to create a potential formulation for oral administration [55] (Table 1). In this study addressing the in vivo antileishmanicidal efficacy of their oral formulation, the authors found that their nanovector delivery system, known as f-CNT–AmB, was able to inhibit the parasite load within the spleen in a dose-dependent manner with 90.2%, 96.5%, and 98.2% inhibition for 5 mg/kg, 10 mg/kg, and 15 mg/kg doses, respectively [55]. In addition, in this small study using a hamster model of infection at the highest oral dose of f-CNT–AmB at 15 mg/kg, it demonstrated comparable efficacy to a 5 mg/kg dose of interperitoneally administered liposomal AmB. Furthermore, the lowest administered dose of their oral formulation (5 mg/kg) had greater efficacy than the same dose of a currently marketed oral treatment for VL, namely miltefosine [55]. Previous work published by these researchers reported on the characterization of their formulation as well as the in vitro cytotoxicity (IC_50_ 0.00234 μg/mL compared to 0.03263 μg/mL for AmB, in a macrophage model), and in vivo safety and efficacy of their formulation following intraperitoneal administration in mice and hamster models (no evidence of toxicity and percent suppression of 89.8% for f-CNT–AmB compared with 68.9% for AmB) [56].

### 5.11. Cubosomes 

Yang et al. (2012) formulated cubosomes as a lipid-based delivery vehicle for AmB, as they believed that their formulation would be able to overcome the molecule’s major inherent drawback, i.e., poor bioavailability [57] (Table 1). In a small animal model, the authors found no indication of nephrotoxicity following a single dose of the oral AmB-loaded cubosomes at doses of 10–20 mg/kg, as measured by plasma BUN and plasma creatinine concentrations. Pharmacokinetic results determined that the cubosome formulation (10 mg/kg) had increased the oral bioavailability of AmB 285% compared with the oral administration of Fungizone^®^. In contrast with the nephrotoxicity results, a biodistribution study showed that the high dose of AmB-loaded cubosomes (20 mg/kg) demonstrated higher uptake of the drug in the kidneys in comparison with the liver and spleen. The liver and the spleen had the highest uptake of the lower dose of AmB-loaded cubosomes. The authors hypothesized that these results may indicate that their formulation would not be able to reduce the kidney toxicity associated with AmB. However, as previously mentioned, this is contradictory to their findings that neither dose caused an abnormal increase plasma BUN and creatinine concentrations 24 h following oral administration [57].

### 5.12. GCPQ Nanoparticles (Quaternary Ammonium Palmitoyl Glycol Chitosan)

Serrano et al. (2015) encapsulated AmB in quaternary ammonium palmitoyl glycol chitosan (GCPQ) nanoparticles in hopes that this self-assembling nanoparticle forming polymer would improve the oral bioavailability of AmB by exhibiting drug delivery to target organs while bypassing toxicity in nontarget organs [58] (Table 1). In order to test their hypothesis, the authors undertook detailed investigations in murine and canine animal models evaluating the efficacy of their formulation in systemic fungal infections, i.e., candidiasis and aspergillosis, in addition to VL. For all tested disease states, AmB–GCPQ had similar efficacy to the marketed parenteral lipid-based formulation AmBisome^®^. Serrano et al. (2015) found that their formulation improved the dissolution of AmB in simulated gastrointestinal fluid compared to conventional AmB. Pharmacokinetic studies showed that AmB–GCPQ delivered more drug to the target organs of pathology, namely, the liver, lung, and spleen, with relatively less delivered to the kidneys. Moreover, the formulation also delivered AmB to the bone marrow and the brain, which the authors argued would be beneficial for the clearance of the *Leishmania* parasite and the treatment of systemic infections, respectively. The reported relative oral bioavailability of the formulation was 24.7% [58].

The aforementioned studies have developed promising formulations which offer a wide range of diverse approaches to overcome the limitations in the development of a viable oral AmB formulation. However, to the best of our knowledge, these formulations have not progressed into the clinical trial stage of development. Conversely, the following two formulations to be discussed are the furthest in the advancement towards achieving the ultimate goal of developing an oral formulation of AmB and bringing it to the market, as they have successfully commenced clinical trials.

### 5.13. Cochleates

Zarif et al. (2008) published results from multiple investigations into the in vitro and in vivo safety and efficacy of their formulation which utilized lipid-based cochleates as a delivery system for AmB for use in *Candida* infections [59] (Table 1). The cochleates consist of solid lipid bilayers arranged in rolled-up sheets that are composed of phospholipid-cation precipitates, specifically phosphatidylserine and calcium, respectively [59]. The AmB encapsulated in the cochleates is thus protected from degradation in the GIT, permitting their use as an oral delivery system. The amphotericin B cochleates (CAMB) formulation was prepared using a hydrogel method and was found to be stable; no drug was lost from the delivery system for four months when stored at 4 °C. In murine models, biodistribution studies provided evidence that absorption through the GI mucosa had occurred, permitting adequate amounts of AmB to reach the target organs affected by systemic fungal infections (i.e., lungs, liver, spleen, and kidneys) following a 10-day oral administration of CAMB (10 mg/kg). The authors believe this absorption occurred due to the involvement of the gut associated lymphatic tissue (GALT), as a large concentration of AmB was found in the liver and spleen. The in vivo studies performed in murine models of *Candida albicans* infection demonstrated that at 0.5 mg/kg/day (up to 2.5 mg/kg/day), CAMB resulted in 100% survival 16 days post-infection compared to 30% mortality in mice treated with 1 mg/kg/day of parenteral Fungizone^®^ (however, 2 mg/kg/day resulted in 100% survival), and 10% mortality resulted from 10 mg/kg/day AmBisome^®^. In addition, CAMB appeared to have comparable efficacy at 2.5 mg/kg/day with that of parenteral Fungizone^®^ at 2 mg/kg/day, resulting in 3.5 log CFU count reduction in the kidneys and no detectable CFUs present in the lungs [59]. In vitro safety studies found no hemolytic effect of CAMB at concentrations up to 500 µg/mL AmB on RBCs. In vivo safety investigations found no abnormal changes in BUN levels and histopathology following 14 day treatment of 50 mg/kg doses of CAMB [59]. Further investigations include the in vivo efficacy in a murine model of *Aspergillus* infection [60]; the in vitro activity in *Leishmania chagasi*; and toxicokinetic studies in vivo in both rat and dog models [61]. All these studies had promising results, which resulted in approval for human trials on CAMB, now known as MAT2203, which is being investigated for the prevention of invasive fungal infections in patients with acute lymphoblastic leukemia [62]. Preliminary results from the Phase I study evaluating the safety, efficacy, tolerability and pharmacokinetics (PK) of CAMB in healthy volunteers has been released [63], demonstrating the potential use of the formulation in single doses of 200 and 400 mg. This study found these doses to be well tolerated with no serious adverse events or laboratory abnormalities and predictable plasma levels comparable to previous animal studies, thus providing evidence that will progress this formulation to the Phase II efficacy trials [63].

### 5.14. SEDDS (iCo-010/019) 

Wasan et al. have also worked to solve the seemingly impossible task of developing an oral AmB formulation for many years [44]. Their approach was to develop a lipid-based self-emulsifying drug delivery system (SEDDS) for AmB to permit oral administration of this poorly bioavailable drug with an additional aim of lessening its nephrotoxicity while maintaining optimal antileishmanial activity [44,64,65,66]. The authors employed mono- and di-glycerides in addition to D-alpha-tocopheryl poly(ethylene glycol) succinate (vitamin E–TPGS). An additional goal was to provide stability for AmB in their delivery system in order to withstand tropical temperatures, considering the clinical target [66]. Before deciding on the iCo-010 formulation, which has recently completed Phase I clinical trials, many versions of the formulation were developed and tested for stability, safety, and efficacy [44,66]. iCo-010 was determined to be the most promising formulation with optimal stability (>75% over 60 days in 30 °C; >95% after 4 h in SIF) antileishmanial activity was observed in a murine model of VL, where <99% reduction in parasitic infection was achieved following 5 days of treatment with 10 mg/kg po bid and 95% inhibition following treatment with 20 mg/kg po qd for 5 days, relative to the control. This formulation also exhibited more desirable self-emulsifying properties compared to other versions of the formulation, namely, iCo-011, -012, -013 [66] (Table 1). The authors hypothesized that the desirable efficacy of their oral AmB formulation was likely a result of improved solubility, stability in the gastrointestinal tract, membrane permeability, and its ability to target the lymphatic transport system. The latter improvement may permit this formulation to target the greatest sites of infection in VL-infected organisms [66]. iCo-10 was also found to maintain AmB in monomeric form upon emulsification in simulated gastric fluid (Wasan lab, unpublished data). Further investigation into the safety of the iCo-010 formulation found no evidence of GI toxicity, hepatotoxicity, or nephrotoxicity following the oral administration of multiple doses in a murine model [65]. The biodistribution of the formulation in a mouse model showed uptake in the organs of the reticuloendothelial system at levels above the IC_50_ for the *leishmania* organism [65], which propelled iCo-010 into Phase I clinical trials. Furthermore, the potential use of iCo-010 for indications other than VL was explored, e.g., systemic candidiasis, which was found to be an effective once daily 5 day treatment for this indication in a rat model [67]. On June 27th, 2018, iCo Therapeutics announced a positive clinical outcome, as the primary safety and tolerability endpoint was met in the Phase I clinical trials of this oral AmB formulation, now known as iCo-019, further supporting the potential of iCo-019 to make it to the market and become accessible to those most affected by VL in endemic regions to have a safe and effective treatment with an oral form of AmB [68].

## 6. Discussion and Concluding Remarks 

The abundance of data published on the topic of developing an oral form of AmB for the treatment of systemic infections such as VL alone supports the urgent need for a formulation to make it to the market. A number of researchers felt inclined to find a solution to overcome the barriers imposed by physicochemical properties of AmB. However, the majority of these formulations were unsuccessful, which demonstrates the difficulty of this task. Nevertheless, the two formulations which have made it to clinical trials with positive preliminary results provide us with evidence that a solution may finally be found which absolves the myth that an oral AmB formulation could not be developed. 

## Figures and Tables

**Table 1:** AmB oral formulation summary.

AmB oral formulation	Efficacy	Stability
Solid lipid nanoparticle [47]	Lower kidney tissue concentration,	2–8 °C for 3 months,
	105% Fo of Fungizone^®^	15 days ≥ 25 °C
PLGA–PEG nanoparticle [43,48]	Increase antifungal activity 4-fold in vitro	N/A
	Inhibit parasite load by 93.2% compared with free AmB group (74.6%)	
	130% Fo of Fungizone^®^	
Chitosan-coated nanostructured lipid carriers [42]	N/A	63.9% AmB retained encapsulated after 30 min incubation in SIF
Lecithin-based mixed polymeric micelles [49]	Less toxic in HT29 cells	Increase solubility
	150% Fo of Fungizone^®^	
O/W microemulsion [50]	Slightly less toxic than free DMSO	Increase the solubility by 1000 folds
Pickering emulsion [51]	N/A	Stable one month under refrigeration
Tragacanth/acrylic acid copolymer [52]	No mortality observed in mice comparing with free AmB	N/A
	Improve oral bioavailability comparing with free AmB	
Chitosan and porphyrin polymeric nanocarrier [53]	23-fold antifungal activity than Ambisome^®^	Less degradation in SIF and a superior release profile for up to 12 h
	Slightly less toxic than Fungizone^®^	
Chitosan–EDTA microparticles [54]	N/A	12-fold improvement in in vitro dissolution relative to pure AmB
Carbon Nanotubes [55,56]	Inhibit the parasite load in a dose-dependent manner	N/A
	No evidence of toxicity in mice and hamster models	
Cubosomes (cubic liquid crystal nanoparticles) [57]	low dose of AmB-loaded cubosomes shows low kidney concentration than Fungizone^®^	74% detectable AmB after 3h in SIF
	285% bioavailability of Fungizone^®^	
GCPQ nanoparticles [58]	Absolute Fo is 24.7%	Stable for a year on storage
	Higher concentration in liver, lung and spleen	
Cochleate–CAMB/MAT2203 [59,60]	100% survival comparing with Fungizone^®^ and AmBisome^®^	Stable for 4 months at 4 °C
	No serious adverse event in Phase I study	
SEDDS (iCo-010/019) [66]	<99% reduction in parasitic infection in a murine model	>75% over 60 days in 30 °C; >95% after 4 h in SIF
	95% inhibition when compared to control	

Abbreviations: SIF, simulated intestinal fluid; Fo, oral bioavailability.

## Abbreviations

Acrylic acid	AAc	Anhydrogalactose	AGR
Amphotericin B	AmB	Blood urea nitrogen	BUN
Amphotericin B cochleates	CAMB	Colony forming units	CFU
Chitosan	CS	1,2-distearoyl-*sn*-glycero-3-phosphoethanolamine- N-methoxy(poly(ethylene glycol)-2000	DSPE-PEG 2K
Carbon nanotubes	CNTs	Ethylenediaminetet-raacetic acid	EDTA
Gut associated lymphatic tissue	GALT	Oral bioavailability	Fo
Hydrophilic–lipophilic balances	HLBs	Gastrointestinal tract	GIT
Oil-in-water microemulsion	O/W ME	Minimum inhibitory concentration	MIC
Polyelectrolyte complexation	PEC	Poly(lactide-*co*-glycolide)–poly(ethylene glycol)	PLGA–PEG
Phamacokinetics	PK	Porphyrin	POR
Quaternary ammonium palmitoyl glycol chitosan	GCPQ	Reactive oxygen species	ROS
Self-emulsifying drug delivery system	SEDDS	Simulated intestinal fluid	SIF
Solid lipid nanoparticles	SLNs	Visceral leishmaniasis	VL

## References

[1] Cereghetti D.M., Carreira E.M. (2006). Amphotericin B: 50 years of chemistry and biochemistry. Synthesis.

[2] Fernandez-Garcia R., de Pablo E., Ballesteros M.P., Serrano D.R. (2017). Unmet clinical needs in the treatment of systemic fungal infections: The role of amphotericin B and drug targeting. Int. J. Pharm..

[3] Mesa-Arango A.C., Scorzoni L., Zaragoza O. (2012). It only takes one to do many jobs: Amphotericin B as antifungal and immunomodulatory drug. Front. Microbiol..

[4] Gray K.C., Palacios D.S., Dailey I., Endo M.M., Uno B.E., Wilcock B.C., Burke M.D. (2012). Amphotericin primarily kills yeast by simply binding ergosterol. Proc. Natl. Acad. Sci. USA.

[5] Kaminski D.M. (2014). Recent progress in the study of the interactions of amphotericin B with cholesterol and ergosterol in lipid environments. Eur. Biophys. J..

[6] Kinsky S.C. (1970). Antibiotic interaction with model membranes. Annu. Rev. Pharmacol..

[7] Palacios D.S., Dailey I., Siebert D.M., Wilcock B.C., Burke M.D. (2011). Synthesis-enabled functional group deletions reveal key underpinnings of amphotericin B ion channel and antifungal activities. Proc. Natl. Acad. Sci. USA.

[8] Wu H.-C., Yoshioka T., Nakagawa K., Shintani T., Tsuru T., Saeki D., Shaikh A.R., Matsuyama H. (2018). Preparation of Amphotericin B-Ergosterol structures and molecular simulation of water adsorption and diffusion. J. Membrane Sci..

[9] Grela E., Wieczor M., Luchowski R., Zielinska J., Barzycka A., Grudzinski W., Nowak K., Tarkowski P., Czub J., Gruszecki W.I. (2018). Mechanism of Binding of Antifungal Antibiotic Amphotericin B to Lipid Membranes: An Insight from Combined Single-Membrane Imaging, Microspectroscopy, and Molecular Dynamics. Mol. Pharm..

[10] Anderson T.M., Clay M.C., Cioffi A.G., Diaz K.A., Hisao G.S., Tuttle M.D., Nieuwkoop A.J., Comellas G., Maryum N., Wang S. (2014). Amphotericin forms an extramembranous and fungicidal sterol sponge. Nat. Chem. Biol..

[11] Mesa-Arango A.C., Trevijano-Contador N., Román E., Sánchez-Fresneda R., Casas C., Herrero E., Argüelles J.C., Pla J., Cuenca-Estrella M., Zaragoza O. (2014). The production of reactive oxygen species is an universal action mechanism of Amphotericin B against pathogenic yeasts and contributes to the fungicidal effect of this drug: AMPHORES study. Antimicrob. Agents Chemother..

[12] Guirao-Abad J.P., Sánchez-Fresneda R., Alburquerque B., Hernández J.A., Argüelles J.-C. (2017). ROS formation is a differential contributory factor to the fungicidal action of amphotericin B and micafungin in Candida albicans. Int. J. Med. Microbiol..

[13] Phillips A.J., Sudbery I., Ramsdale M. (2003). Apoptosis induced by environmental stresses and amphotericin B in Candida albicans. Proc. Natl. Acad. Sci. USA.

[14] Serrano D.R., Lalatsa A. (2017). Oral amphotericin B: The journey from bench to market. J. Drug Deliv. Sci. Tech..

[15] World Health Organization Neglected Tropical Diseases. https://www.who.int/neglected_diseases/diseases/en/.

[16] Global Health Observatory Data Repository Status of Endemicity of Visceral Leishmaniasis Data by Country. http://apps.who.int/gho/data/view.main.NTDLEISHVENDv.

[17] Thornton S.J., Wasan K.M., Piecuch A., Lynd L.L.D., Wasan E.K. (2010). Barriers to treatment for visceral leishmaniasis in hyperendemic areas: India, Bangladesh, Nepal, Brazil and Sudan. Drug Dev. Ind. Pharm..

[18] World Health Organization Leishmaniasis: Epidemiological Situation. https://www.who.int/leishmaniasis/burden/en/.

[19] Global Health Observatory Data Repository Number of Cases of Visceral Leishmaniasis Reported Data by Country. http://apps.who.int/gho/data/node.main.NTDLEISHVNUM?lang=en.

[20] Torres-Guerrero E., Quintanilla-Cedillo M.R., Ruiz-Esmenjaud J., Arenas R. (2017). Leishmaniasis: A review. F1000Research.

[21] Maroli M., Feliciangeli M.D., Bichaud L., Charrel R.N., Gradoni L. (2013). Phlebotomine sandflies and the spreading of leishmaniases and other diseases of public health concern. Med. Vet. Entomol..

[22] Walker D.M., Oghumu S., Gupta G., McGwire B.S., Drew M.E., Satoskar A.R. (2014). Mechanisms of cellular invasion by intracellular parasites. Cell Mol. Life Sci..

[23] Mosser D.M., Edelson P.J. (1987). The third component of complement (C3) is responsible for the intracellular survival of Leishmania major. Nature.

[24] Kima P.E. (2007). The amastigote forms of Leishmania are experts at exploiting host cell processes to establish infection and persist. Int. J. Parasitol..

[25] Beattie L., Kaye P.M. (2011). Leishmania–host interactions: What has imaging taught us?. Cell Microbiol..

[26] Bates P.A. (2018). Revising Leishmania’s life cycle. Nat. Microbiol..

[27] Thornton S.J., Wasan K.M. (2009). The reformulation of amphotericin B for oral administration to treat systemic fungal infections and visceral leishmaniasis. Expert Opin. Drug Deliv..

[28] Grace E., Asbill S., Virga K. (2015). Naegleria fowleri: Pathogenesis, diagnosis, and treatment options. Antimicrob. Agents Chemother..

[29] Belkherroubi-Sari L., Adida H., Seghir A., Boucherit Z., Boucherit K. (2013). New strategy for enhancing the therapeutic index of Fungizone((R)). J. Mycol. Med..

[30] Pham T.T., Loiseau P.M., Barratt G. (2013). Strategies for the design of orally bioavailable antileishmanial treatments. Int. J. Pharm..

[31] Stone N.R., Bicanic T., Salim R., Hope W. (2016). Liposomal amphotericin B (AmBisome®): A review of the pharmacokinetics, pharmacodynamics, clinical experience and future directions. Drugs.

[32] Lister J. (1996). Amphotericin B Lipid Complex (Abelcet) in the treatment of invasive mycoses: The North American experience. Eur. J. Haematol. Suppl..

[33] Clemons K.V., Stevens D.A. (2004). Comparative efficacies of four amphotericin B formulations—Fungizone, Amphotec (Amphocil), AmBisome, and Abelcet—against systemic murine aspergillosis. Antimicrob. Agents Chemother..

[34] Sundar S., Pandey K., Thakur C.P., Jha T.K., Das V.N., Verma N., Lal C.S., Verma D., Alam S., Das P. (2014). Efficacy and safety of amphotericin B emulsion versus liposomal formulation in Indian patients with visceral leishmaniasis: A randomized, open-label study. PLoS Negl. Trop. Dis..

[35] World Health Organization (2010). Control of the leishmaniases. World Health Organ. Tech. Rep. Ser..

[36] Sundar S., Chakravarty J. (2010). Liposomal amphotericin B and leishmaniasis: Dose and response. J. Glob. Infect. Dis..

[37] Sundar S., Chakravarty J. (2015). An update on pharmacotherapy for leishmaniasis. Expert Opin. Pharmacother..

[38] Alvar J., Velez I.D., Bern C., Herrero M., Desjeux P., Cano J., Jannin J., den Boer M., WHO Leishmaniasis Control Team (2012). Leishmaniasis worldwide and global estimates of its incidence. PLoS ONE.

[39] World Health Organization (2010). Costs of Medicines in Current Use for the Treatment of Leishmaniasis. https://www.who.int/leishmaniasis/research/978_92_4_12_949_6_Annex6.pdf?ua=1.

[40] de Assis T.S., Rosa D.C., de Morais Teixeira E., Cota G., Azeredo-da-Silva A.L., Werneck G., Rabello A. (2017). The direct costs of treating human visceral Leishmaniasis in Brazil. Rev. Soc. Bras. Med. Trop..

[41] Jensen G.M. (2017). The care and feeding of a commercial liposomal product: Liposomal amphotericin B (AmBisome((R))). J. Liposome Res..

[42] Ling Tan J.S., Roberts C.J., Billa N. (2018). Mucoadhesive chitosan-coated nanostructured lipid carriers for oral delivery of amphotericin B. Pharm. Dev. Technol..

[43] Radwan M.A., AlQuadeib B.T., Siller L., Wright M.C., Horrocks B. (2017). Oral administration of amphotericin B nanoparticles: Antifungal activity, bioavailability and toxicity in rats. Drug Deliv..

[44] Wasan E.K., Bartlett K., Gershkovich P., Sivak O., Banno B., Wong Z., Gagnon J., Gates B., Leon C.G., Wasan K.M. (2009). Development and characterization of oral lipid-based amphotericin B formulations with enhanced drug solubility, stability and antifungal activity in rats infected with Aspergillus fumigatus or Candida albicans. Int. J. Pharm..

[45] Barwicz J., Tancrede P. (1997). The effect of aggregation state of amphotericin-B on its interactions with cholesterol- or ergosterol-containing phosphatidylcholine monolayers. Chem. Phys. Lipids.

[46] Espada R., Valdespina S., Alfonso C., Rivas G., Ballesteros M.P., Torrado J.J. (2008). Effect of aggregation state on the toxicity of different amphotericin B preparations. Int. J. Pharm..

[47] Chaudhari M.B., Desai P.P., Patel P.A., Patravale V.B. (2016). Solid lipid nanoparticles of amphotericin B (AmbiOnp): In vitro and in vivo assessment towards safe and effective oral treatment module. Drug Deliv. Transl. Res..

[48] Kumar R., Sahoo G.C., Pandey K., Das V., Das P. (2015). Study the effects of PLGA-PEG encapsulated amphotericin B nanoparticle drug delivery system against Leishmania donovani. Drug Deliv..

[49] Chen Y.C., Su C.Y., Jhan H.J., Ho H.O., Sheu M.T. (2015). Physical characterization and in vivo pharmacokinetic study of self-assembling amphotericin B-loaded lecithin-based mixed polymeric micelles. Int. J. Nanomed..

[50] Silva A.E., Barratt G., Cheron M., Egito E.S. (2013). Development of oil-in-water microemulsions for the oral delivery of amphotericin B. Int. J. Pharm..

[51] Richter A.R., Feitosa J.P.A., Paula H.C.B., Goycoolea F.M., de Paula R.C.M. (2018). Pickering emulsion stabilized by cashew gum- poly-l-lactide copolymer nanoparticles: Synthesis, characterization and amphotericin B encapsulation. Colloids Surf. B Biointerfaces.

[52] Mohamed H.A., Radwan R.R., Raafat A.I., Ali A.E. (2018). Antifungal activity of oral (Tragacanth/acrylic acid) Amphotericin B carrier for systemic candidiasis: In vitro and in vivo study. Drug Deliv. Transl. Res..

[53] Bhatia S., Kumar V., Sharma K., Nagpal K., Bera T. (2014). Significance of algal polymer in designing amphotericin B nanoparticles. Sci. World J..

[54] Singh K., Tiwary A., Rana V. (2013). Spray dried chitosan–EDTA superior microparticles as solid substrate for the oral delivery of amphotericin B. Int. J. Biol. Macromol..

[55] Prajapati V.K., Awasthi K., Yadav T.P., Rai M., Srivastava O.N., Sundar S. (2011). An oral formulation of amphotericin B attached to functionalized carbon nanotubes is an effective treatment for experimental visceral leishmaniasis. J. Infect. Dis..

[56] Prajapati V.K., Awasthi K., Gautam S., Yadav T.P., Rai M., Srivastava O.N., Sundar S. (2011). Targeted killing of Leishmania donovani in vivo and in vitro with amphotericin B attached to functionalized carbon nanotubes. J. Antimicrob. Chemother..

[57] Yang Z., Tan Y., Chen M., Dian L., Shan Z., Peng X., Wu C. (2012). Development of amphotericin B-loaded cubosomes through the SolEmuls technology for enhancing the oral bioavailability. AAPS PharmSciTech.

[58] Serrano D.R., Lalatsa A., Dea-Ayuela M.A., Bilbao-Ramos P.E., Garrett N.L., Moger J., Guarro J., Capilla J., Ballesteros M.P., Schatzlein A.G. (2015). Oral particle uptake and organ targeting drives the activity of amphotericin B nanoparticles. Mol. Pharm..

[59] Zarif L., Graybill J.R., Perlin D., Mannino R.J. (2000). Cochleates: New lipid-based drug delivery system. J. Liposome Res..

[60] Delmas G., Park S., Chen Z.W., Tan F., Kashiwazaki R., Zarif L., Perlin D.S. (2002). Efficacy of orally delivered cochleates containing amphotericin B in a murine model of aspergillosis. Antimicrob. Agents Chemother..

[61] Kalbag S., Lu R., Ngoje J., Mannino R.J. (1992). Oral Administration of Amphotericin B: Toxicokinetic Studies in Animal Models. Antimicrob. Agents Chemother..

[62] Matinas Biopharma MAT2203: LNC Formulation of Amphotericin B. https://www.matinasbiopharma.com/pipeline/mat2203-lnc-formulation-of-amphotericin-b.

[63] Mannino R., De B., Teae A. Oral Administration of Amphotericin B (CAmB) in Humans: A Phase I Study of Tolerability and Pharmacokinetics Preliminary Pharmacokinetics. https://content.equisolve.net/_db6027646f523d19fe795801a0b7aff1/matinasbiopharma/db/128/510/pdf/Oral_Dosing_of_Encochleated_Amphotericin_B_%28CAmB%29__Rapid_Drug_Targeting_to_Infected_Tissues_in_Mice_with_Invasive_Candidiasis.pdf.

[64] Wasan K.M., Wasan E.K., Gershkovich P., Zhu X., Tidwell R.R., Werbovetz K.A., Clement J.G., Thornton S.J. (2009). Highly effective oral amphotericin B formulation against murine visceral leishmaniasis. J. Infect. Dis..

[65] Sivak O., Gershkovich P., Lin M., Wasan E.K., Zhao J., Owen D., Clement J.G., Wasan K.M. (2011). Tropically stable novel oral lipid formulation of amphotericin B (iCo-010): Biodistribution and toxicity in a mouse model. Lipids Health Dis..

[66] Wasan E.K., Gershkovich P., Zhao J., Zhu X., Werbovetz K., Tidwell R.R., Clement J.G., Thornton S.J., Wasan K.M. (2010). A novel tropically stable oral amphotericin B formulation (iCo-010) exhibits efficacy against visceral Leishmaniasis in a murine model. PLoS Negl. Trop. Dis..

[67] Ibrahim F., Sivak O., Wasan E.K., Bartlett K., Wasan K.M. (2013). Efficacy of an oral and tropically stable lipid-based formulation of Amphotericin B (iCo-010) in an experimental mouse model of systemic candidiasis. Lipids Health Dis..

[68] Rae A. (2018). iCo Therapeutics Announces Positive Oral Amphotericin Study. https://ceo.ca/@newsfile/ico-therapeutics-announces-positive-clinical-outcome.

